# The (C_2_N_2_H_10_)[Cu(H_2_O)_4_](*TX*_4_)_2_ Structural Family: When Fluoroberyllate, Sulfate, and Selenate Are Full Analogs

**DOI:** 10.3390/molecules29225372

**Published:** 2024-11-14

**Authors:** Dmitri O. Charkin, Vadim E. Kireev, Dmitri N. Dmitriev, Alexander M. Banaru, Alena A. Kompanchenko, Dina V. Deyneko, Ivan G. Tananaev, Sergey M. Aksenov

**Affiliations:** 1 Department of Chemistry, Moscow State University, 1-3 Leninskie Gory, Moscow 199991, Russia; d.o.charkin@gmail.com (D.O.C.); ddn063@gmail.com (D.N.D.); banaru@mail.ru (A.M.B.); deynekomsu@gmail.com (D.V.D.); 2 Laboratory of Arctic Mineralogy and Material Sciences, FRC Kola Science Centre RAS, 14 Fersman Str., Apatity 184209, Russia; kvad2000@yandex.ru; 3 Geological Institute, FRC Kola Science Centre RAS, 14 Fersman Str., Apatity 184209, Russia; komp-alena@yandex.ru; 4 I.V. Tananaev Institute of Chemistry and Technology of Rare Elements and Mineral Raw Materials, FRC Kola Science Centre RAS, 26a, Akademgorodok, Apatity 184209, Russia; geokhi@mail.ru; 5 A.M. Frumkin Institute of Physical Chemistry and Electrochemistry, 31-4 Leninsky Prospect, Moscow 119071, Russia

**Keywords:** fluoroberyllate, selenate, sulfate, solution synthesis, organo-inorganic hybrid structures, topological features, crystal chemistry

## Abstract

Two new organo-inorganic hybrids, (C_2_N_2_H_10_)[Cu(H_2_O)_4_](BeF_4_)_2_ (**1**) and (C_2_N_2_H_10_)[Cu(H_2_O)_4_](SeO_4_)_2_ (**2**), were prepared via the interaction of ethylenediamine, copper fluoroberyllate or selenate, and H_2_[BeF_4_]/H_2_SeO_4_ in aqueous solutions. The structures of **1** and **2** are similar to each other and the previously reported (C_2_N_2_H_10_)[Cu(H_2_O)_4_](SO_4_)_2_: monoclinic, *P*2_1_/*c*, *a* = 5.1044(2) Å, *b* = 11.6171(4) Å, *c* = 10.1178(3) Å, and β = 94.431(3)° for **1**; and *a* = 5.25020(10), *b* = 11.7500(2), *c* = 10.4434(2), and β = 94.5464(17)° for **2**. All structures contain a square planar [Cu(H_2_O)_4_]^2+^ species, which coordinates, at rather long distances, two *TX*_4_^2−^ tetrahedral dianions in κ^1^ mode, forming relatively weak [Cu(H_2_O)_4_(*TX*_4_)_2_]^2−^ complexes. These are linked together via hydrogen bonding into pseudo-chains; the ethylenediammonium cations link them into a 3D architecture. Compound **1** is, to the best of our knowledge, the first—though expected—representative of a hybrid organo-inorganic fluoroberyllate. The crystal chemical relations within the structural family (enH_2_)[Cu(H_2_O)_4_](*TX*_4_)_2_ are discussed.

## 1. Introduction

Despite the notorious high toxicity of beryllium compounds [1], these are investigated actively due to a variety of attractive properties which these exhibit with high performance. For instance, berylloborates [2,3] and beryllosulfates [4] demonstrate high nonlinear optical activity. A single example of a fluoroberyllate with large birefringence is reported in [5]. However, since the chemistry of beryllium has not been sufficiently studied due to its toxicity, targeted synthesis of new promising compounds is difficult, and systematic studies are developing mainly around several structural families, in particular, the KBBF-type of layered materials named after potassium beryllium borate fluoride with the chemical formula KBe_2_BO_3_F_2_ [3,6,7,8,9] because of their advanced physical properties. There exists one relatively simple yet numerous family of beryllium compounds, the fluoroberyllates, which exhibit strong structural similarities to sulfates [10,11,12] since the sizes of the [BeF_4_]^2−^ and [SO_4_]^2−^ anions are nearly equal. Fluoroberyllate analogs have been reported for a variety of sulfates and sometime selenates, both simple (e.g., [13]) and more-complex (e.g., [14]) compounds, to cite just a few.

However, to the best of our knowledge, no fluoroberyllate analogs are known for hybrid organo-inorganic sulfates, which are plentiful [15]. The only example is the group of transition metal hydrazinium sulfates and fluoroberyllates, (N_2_H_5_)_2_*M*(SO_4_)_2_ [16] and (N_2_H_5_)_2_*M*[BeF_4_]_2_ [13] (*M* = divalent 3*d*-cation). Based on these widespread analogies, the existence of hybrid organo-inorganic fluoroberyllates is highly likely. Here, we present the first (to the best of our knowledge) example of such a compound, ethylenediammonium tetraaquacopper(2+) bis-tetrafluoroberyllate, (enH_2_)[Cu(H_2_O)_4_][BeF_4_]_2_ (**1**), which is a full chemical analog of the respective sulfate (enH_2_)[Cu(H_2_O)_4_][SO_4_]_2_ [17]. In addition, a selenate analog of these, (enH_2_)[Cu(H_2_O)_4_](SeO_4_)_2_ (**2**), has also been prepared and characterized, and the crystal chemical relations within the structural family (enH_2_)[Cu(H_2_O)_4_](*TX*_4_)_2_ are discussed.

## 2. Results and Discussion

### 2.1. Crystal Structure

**Figure 1:**
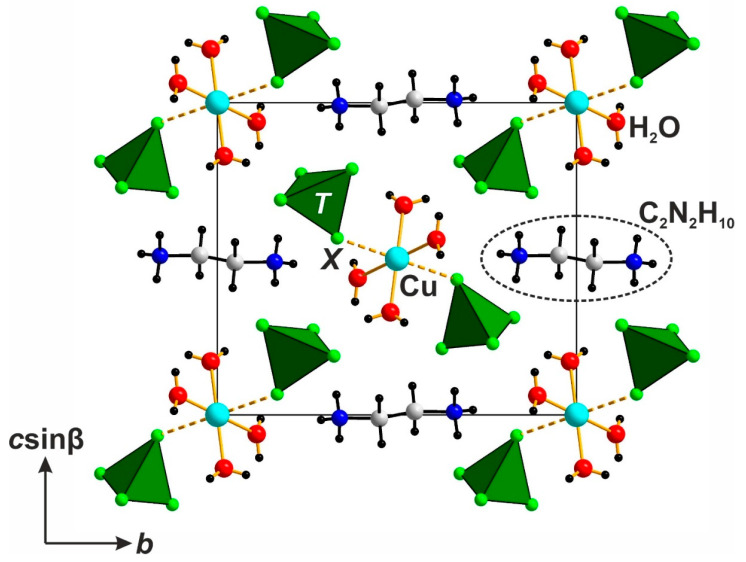
The general view of monoclinic (sp. gr. *P*2_1_/*c*) compounds belonging to (C_2_N_2_H_10_)[Cu(H_2_O)_4_](*TX*_4_)_2_ structural family, where *T* = Be^2+^, S^6+^, Se^6+^; *X* = F^−^, O^2–^.

The crystal structures of both **1** and **2** (Figure 1) are fully analogous to each other and isostructural with the previously reported sulfate, (enH_2_)[Cu(H_2_O)_4_](SO_4_)_2_ [17]. Only the structural data have been provided for the latter; therefore, it is discussed here in more detail in comparison to those of **1** and **2**.

**Figure 2:**
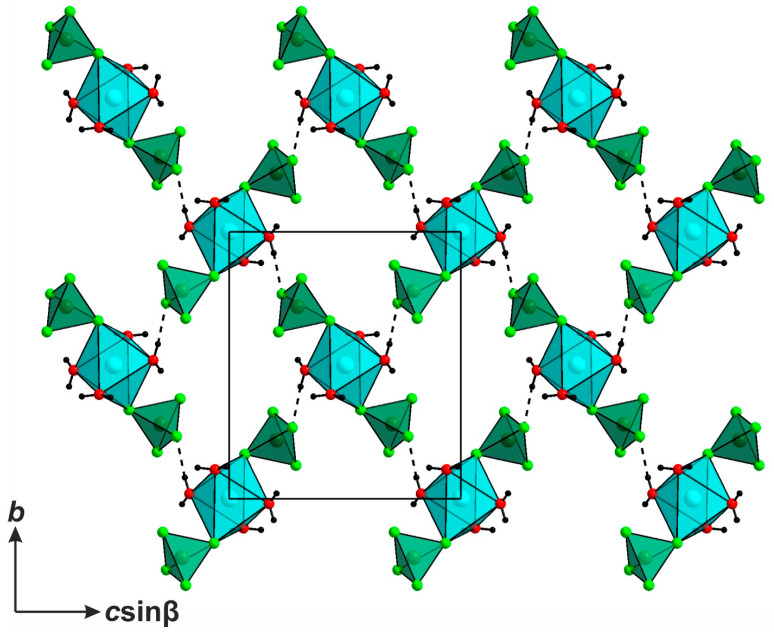
The hydrogen-bonded inorganic framework comprising [Cu(H_2_O)_4_[BeF_4_]_2_]^2−^ complexes. The CuO_4_F_2_ are shown in blue, while the BeF_4_ tetrahedra are shown in green.

The structures of **1** and **2** and their respective sulfate can be considered as comprising [enH_2_]^2+^ cations, square planar [Cu(H_2_O)_4_]^2+^ cations, and *TX*_4_^2−^ tetrahedral anions. The two latter species form relatively weak [Cu(H_2_O)_4_(*TX*_4_)_2_]^2−^ complexes, relatively common for the transition metal sulfates and selenates [18,19,20,21,22,23]—those for **1**, as shown in Figure 1. The complexes are linked by hydrogen bonds between the water molecules of the [Cu(H_2_O)_4_]^2+^ cores and the terminal vertices of the *TX*_4_^2−^ tetrahedra, forming 3D frameworks (Figure 2).

**Figure 3:**
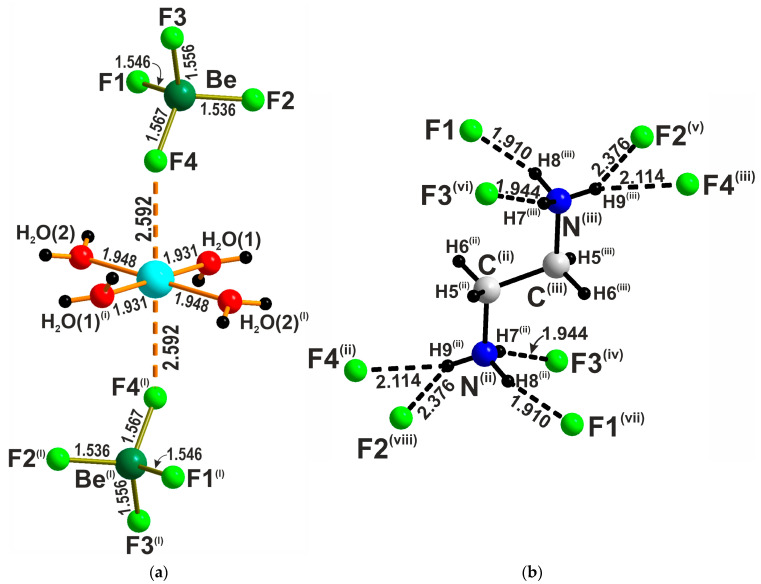
The complex [Cu(H_2_O)_4_[BeF_4_]_2_]^2−^ anion (**a**) and coordination environment of the enH_2_^2+^ cation (**b**) in the crystal structure of **1**. Symmetry codes are 2 − *x*, 1−*y*, 1 − *z* (i); 1 − x, *y* − ½, ½ − *z* (ii); *x* − 1, 1.5 − *y*, *z* − ½ (iii); −*x*, *y* − ½, ½ − *z* (iv); −1 + *x*, *y*, *z* (v); *x*, 1.5 − *y*, *z* − ½ (vi), −*x*, 1 − *y*, −*z* (vii); and 1 − *x*, 1 − *y*, −*z* (viii).

The environment of copper can thus be regarded as a strongly elongated CuO_4_*X*_2_ octahedron with *X* atoms (O in SO_4_^2−^ and SeO_4_^2−^ and F in BeF_4_^2−^) at the apical sites (Figure 3a). This distortion can be explained by significant differences in the chemical nature of the ligands and, probably, by the electronic Jahn–Teller effect typical for Cu^2+^. The value of quadratic elongation [24] calculated in the VESTA ver. 3.5.8 program package [25] λ = 1.0560, 1.0502, and 1.0465 for the CuO_4_*X*_2_ octahedron and λ = 1.0002, 1.0004, 1.0013 for the *TX*_4_ tetrahedron for *T* = S, Se, and Be, respectively. The fractional part of the quadratic elongation parameter λ [24] is approximately equal to twice the percentage deviation of the polyhedron from its holosymmetric configuration. Consequently, in the series *T* = S, Se, and Be, this percentage deviation decreases from ca. 2.80 to 2.32% for the CuO_4_*X*_2_ octahedron, while it slightly increases from ca. 0.01 to 0.06% for the *TX*_4_ tetrahedron. Thus, the closer the square coordination of Cu is to the octahedral one, the more the anion is deformed; thus, the [Cu(H_2_O)_4_]^2+^ core remains nearly invariable.

The axes of the complexes are oriented along (111) and (1-11) in a chess-like order. The (enH_2_)^2+^ cations fill the gaps in the inorganic framework and also form six hydrogen bonds—two of which are bifurcated—to the vertices of the *TX*_4_ tetrahedra (Figure 3b).

### 2.2. Overall Remarks

The new compound **1** is, to the best of our knowledge, the first example of a hybrid organo-inorganic fluoroberyllate besides the small series of compounds containing the organic cations, Li^+^ and [BeF_4_]^2–^ [26,27,28]. In the latter cases, the Li^+^ cations are parts of the metal–fluoride frameworks comprising edge- and vertex-sharing LiF_4_ and BeF_4_ tetrahedra. New compounds of other inorganic cations can therefore be expected to be discovered.

The transition metal-based constituent, the [Cu(H_2_O)_4_]^2+^ moiety, remains nearly invariable in all three structures under discussion; its size is 7.52(4) Å^2^ for **1** and 7.59(4) Å^2^ for **2**, which is virtually constant within the error limits. The size of the tetrahedral anion affects more essentially the volume of the CuO_4_*X*_2_ octahedron (*X* = O or F): 12.994(5) Å^3^ for **1**; 13.515(2) Å^3^ for the sulfate; and 13.135(3) Å^3^ for **2**. Keeping in mind that the volumes of the SO_4_^2−^, BeF_4_^2−^, and SeO_4_^2−^ equal 1.653(4) Å^3^, 1.914(5) Å^3^, and 2.267(6) Å^3^, respectively, we estimate the volumes of the [Cu(H_2_O)_4_(*TX*_4_)_2_]^2−^ complexes as 16.822(5) Å^3^ for **1**, 16.821(4) for the sulfate, and 17.669(4) Å^3^ for **2**. This does not agree with the trend in the cell volumes, which may occur due to the differences in the lengths (hence the strengths) of the hydrogen bonds (Table S2). The N-H···O and O-H···O bonds to the oxyanions are somewhat longer compared to the N-H···F and O-H···F bonds, most likely due to the higher negative change on the fluorine atoms (almost fluoride anions).

When a short contact *M*···*X*–*T* formed by the core ZB = [Cu(H_2_O)_4_] and ZC = *TX*_4_ is taken into consideration along H-bonds, the 6,7,8-coordinated net with point symbol (4^12^.6^3^)(4^15^.6^6^)_2_(4^20^.6^8^) is derived (CN_ZA/ZC_ = 6; CN_ZC/(ZA&ZB)_ = 7; CN_ZB/ZC_ = 8); TD_10_ = 2312. When only H-bonds are taken into consideration, the 6,6-coordinated net with point symbol (4^10^.6^5^)(4^7^.6^8^) is derived (CN_ZA/ZC_ = CN_ZB/ZC_ = CN_ZC/(ZA&ZB)_ = 6; ZA and ZB are equivalent with respect to automorphism group of the net); TD_10_ = 2276. In the TopCryst database [29], this net type is referred to as **6,6T206**, with seven occurrences in structural databases to date.

The space-group type *P*2_1_/*c* has three symmetry operations in any minimal generating subset [30]; i.e., if b.u. occupied a sole general position in the space group of this type, the structure would be of IHD = 3. Actually, in **1** and **2**, Wyckoff positions occupied by ZA, ZB, and ZC are 2*d*, 2*b* (both in inversion centers), and 4*e* (a general position), respectively. The closest inversion centers belonging to 2*d* and 2*b* are separated by *c*/2; thus, a pair of them can generate the translation **c**. However, there is a glide plane *c* going in this direction in the space group, and the above pair of inversion centers cannot generate this glide plane: just one of the two types of inversion is fit for a minimal generating set of the group. Consequently, *f* = 1 (not 2), causing IHD = 3 + 3 − 1 − 1 = 4; i.e., some subset of four H-bonds (out of the six symmetrically independent H-bonds in the structure) would be enough to generate a connected H-bonded net from the b.u., whereas some subset of three H-bonds would not be enough. This judgement is easily tested by deriving all the combinatorially different subnets of the initial net based on triples of non-equivalent H-bonds. Although there are six 3D subnets conforming to this requirement, none of them are simply connected and comprise two interpenetrated subnets.

### 2.3. Comparison to Related Species

**Figure 4:**
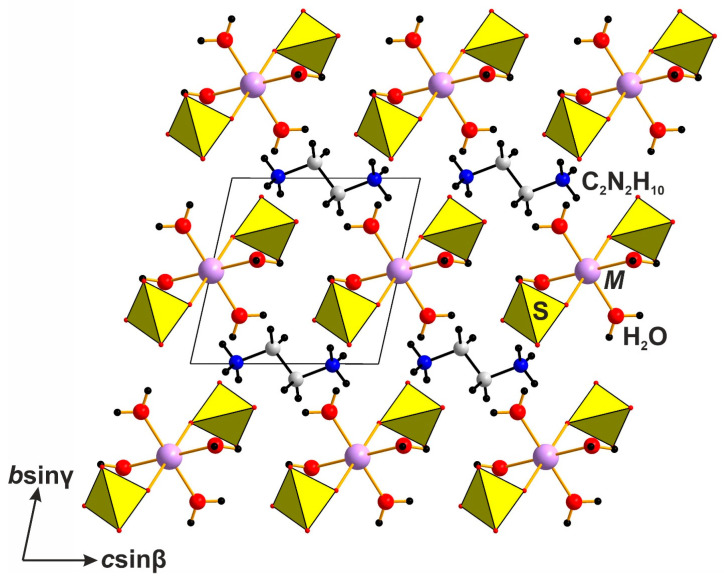
A general view of triclinic compounds with the general formula (enH_2_)[*M*(H_2_O)_4_](SO_4_)_2_, where *M* = Mg, Mn, Fe, Co, and Cd.

**Figure 5:**
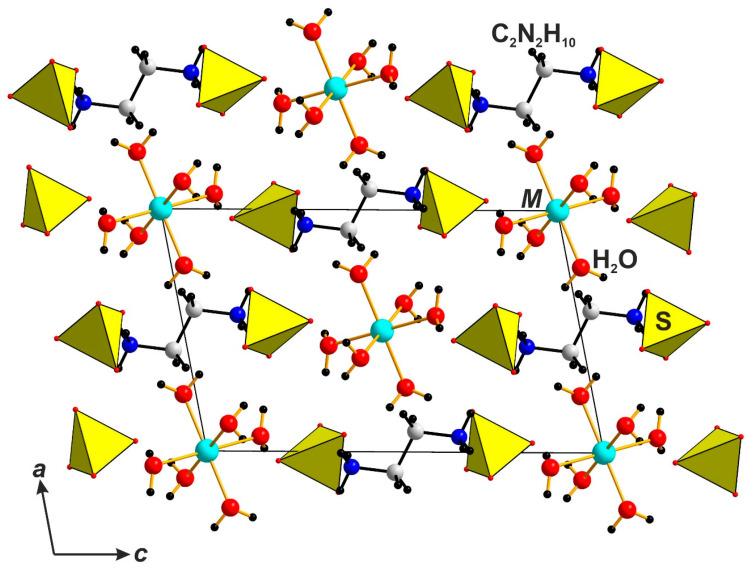
The general view of the crystal structures of hexahydrates with the general formula (enH_2_)[*M*(H_2_O)_6_](SO_4_)_2_] (M = Ni, Zn).

As noted above, double sulfates of organic and transition metal cations are studied more thoroughly, and comparisons will, as yet, be restricted herein to this family of compounds with the general formula (enH_2_)[*M*(H_2_O)_4_(*TX*_4_)_2_]. Double sulfates of the overall (enH_2_)*M*(SO_4_)_2_·4H_2_O compositions (Table 1) have been prepared for a variety of transition metals (Mg, Mn, Co, Cu, and Cd) [19,20,21,22,23]. Tetrahydrates, except for that of Cu, are isostructural. While the majority of these are triclinic (for *M* = Mg, Mn, Fe, Co, and Cd) (Figure 4), that of Cu adopts a higher monoclinic symmetry with the sp.gr. *P*2_1_/*c* (Figure 2). The difference between these structures mostly concerns the arrangement of the [*M*(OH_2_)_4_(SO_4_)_2_]^2−^ complexes. For *M* = Mg, Mn, Co, and Cd; they align parallel to each other along (111); whereas for *M* = Cu, they align in alternating manner along (111) and (1-11). For Ni and Zn sulfates, only the hexahydrates (enH_2_)[Ni(H_2_O)_6_](SO_4_)_2_ [31] and (enH_2_)[Zn(H_2_O)_6_](SO_4_)_2_ [32] were reported. These compounds are characterized by the presence of three types of isolated units (Figure 5): [*M*(H_2_O)_6_]–metal complex (M = Ni, Zn), [SO_4_] tetrahedra, and (enH_2_)^2+^ organic cation, which are linked via a system of hydrogen bonds. Among the related selenates, the monoclinic dihydrate compound (enH_2_)[Cd(H_2_O)_2_(SeO_4_)_2_] [33] is known, which is based on isolated [Cd(H_2_O)_2_(SeO_4_)_2_]-chains of the kröhnkite type [34], which are linked by the hydrogen bonds of (enH_2_)^2+^ cations and water molecules.

**Figure 6:**
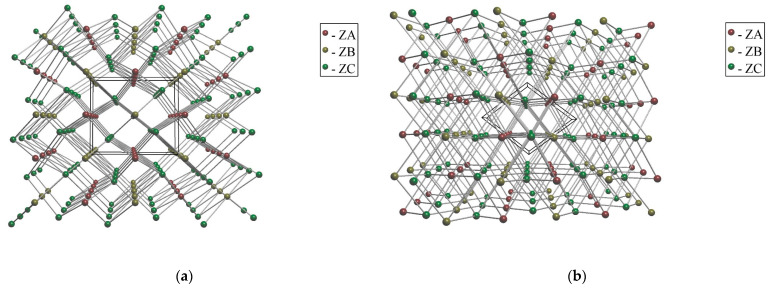
Standard representation of H-bonded nets of (C_2_N_2_H_10_)[*M*(H_2_O)_4_](SO_4_)_2_. Topological types: **6,6T206** for *M* = Cu (**a**); and **6,6T1** for *M* = Co (**b**). ZA = C_2_H_10_N_2_^2+^, ZB = [*M*(H_2_O)_4_]^2+^, ZC = SO_4_^2–^. An edge designate H-bonded pair of b.u.

For the triclinic tetrahydrates (with *M* = Mg, Mn, Co, Cu, and Cd), *M* = Co was taken to exemplify this structural type, with the structural data being extracted from [35] instead of [21] as it contains refined protons (Figure 4). When a short contact *M*···*X*–*T* formed by the core ZB = [*M*(H_2_O)_4_] and ZC = *TX*_4_ is taken into consideration along the H-bonds, the 6-, 7-, 8-coordinated net is again derived (CN_ZA/ZC_ = 6, CN_ZC/(ZA&ZB)_ = 7, CN_ZB/ZC_ = 8 are the same). However, its point symbol—viz. (4^12^.6^3^)(4^18^.6^3^)_2_(4^18^.6^9^.8); TD_10_ = 2203—differs from that obtained for *M* = Cu. When solely H-bonds are taken into consideration, the 6,6-coordinated net is again derived (CN_ZA/ZC_ = CN_ZB/ZC_ = CN_ZC/(ZA&ZB)_ = 6; ZA and ZB are again equivalent with respect to automorphism group of the net). However, its topologic type (4^13^.6^2^)(4^8^.6^6^.8) is different from that for *M* = Cu (Figure 6), and its TD_10_ = 1745 is much less. In the TopCryst database [29], this net type is referred to as **6,6T1** with 62 occurrences in structural databases up to time; i.e., this type is more widespread than the former one. Moreover, this type is now found among those deposited to RCSR [36] with reference code **htp.**

**Figure 7:**
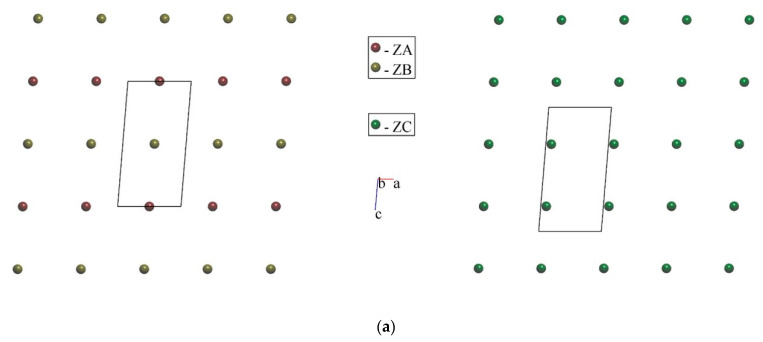

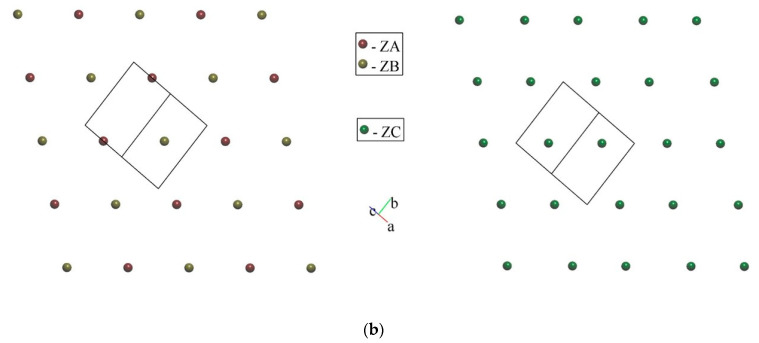
Plane arrangement of ZA = C_2_H_10_N_2_^2+^ and ZB = [*M*(H_2_O)_4_]^2+^ (left side) and ZC = SO_4_^2–^ (right side) for *M* = Cu, view along [010] (**a**), and *M* = Co, view along [101] (**b**).

It should be emphasized that the arrangement of b.u. mass centers in both structural types can be considered as almost-plane sheets filled by ZC alternating with those filled by ZA and ZB. For *M* = Cu, those sheets are stacked along *b*; while for *M* = Co, they are stacked along [101] direction (Figure 7). Within one sheet, ZA and ZB are arranged in chessboard order (a rock-salt arrangement) for *M* = Co but not for *M* = Cu.

The space-group type $P\bar{1}$, contrary to *P*2_1_/*c*, has four symmetry operations in any minimal generating subset [30]. In isostructural tetrahydrates belonging to this space-group type, ZA, ZB, and ZC are occupying Wyckoff positions 1*b*, 1*e* (both in inversion centers again) and 2*i* (a general position). Unlike *P*2_1_/*c*, for $P\bar{1}$ any two closest inversion centers are fit for the same minimal generating set of the group, thus, *f* = 2. Consequently, IHD = 4 + 3 − 2 − 1 = 4 again. Indeed, there are 12 combinatorially different subnets of the initial H-bonded net based on quadruples of non-equivalent H-bonds, each a simply connected one. Thus, despite another structure, all the tetrahydrates are of the same hierarchical complexity evaluated by IHD.

The structures reported here provide a new example of structural similarity between fluoroberyllates, sulfates, and selenates, which is not common as there are just small overlapping areas in the chemistry of complex derivatives of these anions. We note that in the current case, both enH_2_^2+^ and Cu^2+^ form isostructural compounds with all the three anions discussed here [37,38,39,40,41,42]. Before any general predictions and conclusions can be made, systematic studies of derivatives of all three anions should be performed in systems containing similar organic and inorganic cations. However, we foresee formation for BeF_4_^2−^-based analogs of double sulfates and selenates in cases when the coordination sphere of the metal cation is completely or nearly completely filled by water molecules or some other ligands. Studies are currently underway to check the consistency of these suggestions.

## 3. Experimental Section

### 3.1. Synthesis

*Caution! Beryllium compounds are highly toxic and carcinogenic. All manipulations should be performed in a properly equipped laboratory by a trained personnel.*

The preparation of the sulfate and selenate compounds followed the common procedure of the room-temperature evaporation of aqueous solutions containing en, Cu*T*O_4_, and H_2_*T*O_4_ (*T* = S or Se) in a 1:1:1.05 molar ratio. The fluoroberyllate was prepared in several steps according to [43]. First, 900 mg (100 mmol) of beryllium powder was added to a solution containing 16 mL 40% HF (400 mmol) and 50 mL of distilled water. The solution was then diluted to 100 mL to yield 1 *M* H_2_[BeF_4_]. Next, copper hydroxide carbonate was introduced until no more carbon dioxide evolved. *Both steps should be undertaken in very small portions under strong cooling and mild stirring to prevent effervescence and formation of hazardous beryllium-containing aerosols. Covering of the reaction vessel is strongly advised*. The blue solution thus formed was separated from the unreacted copper carbonate and allowed to evaporate at room temperature. Crystallization of Cu[BeF_4_]·5H_2_O was nearly complete in 10 days. As the crystals of the copper fluoroberyllate pentahydrate readily effloresce in air, similar to the isostructural sulfate and selenate, these were stored in a closed PP vial together with several drops of the mother liquor. To prepare the target compound **1**, a small crystal of Cu[BeF_4_]·5H_2_O was dissolved in a calculated aliquot of 1 *M* H_2_[BeF_4_], to which one drop of 40% HF was added. A corresponding amount of en was neutralized by H_2_[BeF_4_]; the two transparent solutions were mixed and also left evaporate at room temperature until crystallization. As the target compounds are also prone to efflorescence, these were kept in closed PP vials under mother liquors prior to analyses.

### 3.2. Crystal Structure Determination

Single crystals of the new compounds (enH_2_)[Cu(H_2_O)_4_][BeF_4_]_2_ (**1**) and (enH_2_)[Cu(H_2_O)_4_](SeO_4_)_2_ (**2**) were chosen under polarization microscope. X-ray data were collected at room temperature on a XtaLAB Synergy diffractometer (Rigaku, Tokyo, Japan) (Mo*K*α radiation, Hybrid Pixel Array detector). The CrysAlis software package [44] was used to determine and refine unit-cell parameters using least-square techniques, to integrate data, and to correct for background, Lorentz, and polarization effects. Experimental details are collected in Table 2.

Based on the analysis of the systematic absences compounds **1** and **2** are both characterized by centrosymmetric space group *P*2_1_/*c*. The structure models were derived via the “charge-flipping” method using SUPERFLIP program [45]. The refinements were performed using Jana2006 [46] and Jana2020 [47]. The hydrogen atoms of the water molecules were located on a difference Fourier map. Initial illustrations were produced with the Jana2020 program package in combination with DIAMOND [48].

The structures were refined anisotropically. The selected bond distances for **1** and **2** are given in Table S1 (Supplementary Materials), respectively. Geometrical features of hydrogen bonds are listed in Tables S2. CCDC 2390641 and 2390642 contain supplementary crystallographic data for compounds **1** and **2**, respectively. The data can be obtained free of charge from The Cambridge Crystallographic Data Centre via www.ccdc.cam.ac.uk/structures.

### 3.3. Raman Spectroscopy

**Figure 8:**
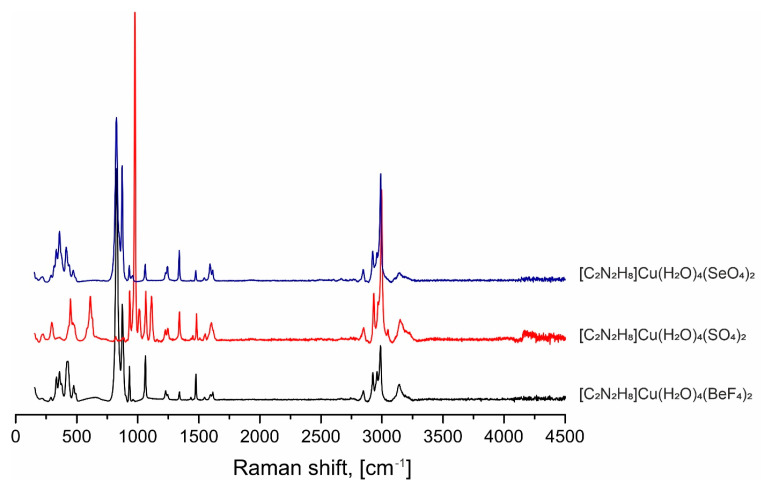
The Raman spectra of compounds of compounds (enH_2_)[Cu(H_2_O)_4_(*TX*_4_)_2_] structural family.

The spectra were registered using an EnSpectr R532 spectrometer (EnSpectr, Meridian, MS, USA) combined with an Olympus BX-43 optical microscope (Olympus, Tokyo, Japan). Raman spectra were excited using a 532 nm solid-state laser with an actual power of 18 mW under the 100× objective (NA 0.8). Registration range: 150–4500 cm^−1^; resolution: 5–8 cm^−1^; exposition time: 1 sec; and the number of acquisitions: 30; precision ±1 cm^−1^; laser beam diameter: ca. 2 mm. The experimental Raman spectra for compounds (enH_2_)[Cu(H_2_O)_4_(*TX*_4_)_2_] are shown in Figure 8 and in Table 3.

As follows from Figure 8, the Raman spectra are very similar for all three compounds (enH_2_)[Cu(H_2_O)_4_(*TX*_4_)_2_], which is evident due to the closeness of their composition. The position and intensities of the bands, as well as their tentative assignments, performed based on data given in [49,50,51,52,53,54] are summarized in Table 3. The positions of most bands corresponding to the [Cu(H_2_O)_4_]^2+^ flat square base of the distorted Cuφ_6_-octahedra (φ = O^2–^, F^−^, H_2_O^0^), as well as to the organic cations, are nearly invariable. The main differences are due to the nature of the tetrahedral anions, yet there are essential overlaps between the bands.

### 3.4. Topological Analysis

The atomic adjacency matrix was constructed by the domain method [55]. In order to simplify structural model, the standard topological representation [56] for all the structures under consideration was used. All calculations were performed in ToposPro program package [57] ver. 5.4.3.0. To build a standard representation of a structure with a 0D building unit (b.u.) and intra- and intermolecular contacts the groups of atoms are united by intramolecular contacts into centroids, while the intermolecular contacts are transformed into the edges of the underlying net. The underlying net characterizes the packing of 0D b.u., e.g., complex ions and aqua molecules. Herein, to form the edges of the underlying net, only H-bonds and shor-contacts *M*···*X*–*T* were considered, and the set of 0D b.u. was the following: ZA = C_2_H_10_N_2_^2+^; ZB = [*M*(H_2_O)_4_]^2+^; ZC = *TX*_4_^2–^.

The derived nets were distinguished with the aid of TopCryst database [29] by **NDn** nomenclature [58], where **N** is a sequence of coordination numbers of all non-equivalent nodes of the net, **D** is the periodicity of the net, and **n** is the ordinal number of the net in the set of all non-isomorphic nets with the given **ND** sequence. The nets that were not classified in these databases were characterized by a point symbol [59]: the notation *A^a^*.*B^b^*… means that *a* angles belonging to the shortest *A*-gonal cycle, *b* angles belonging to the shortest *B*-gonal cycle, etc., are converging at the vertex of the net, while *A* < *B* < … and *a* + *b* + … = CN(CN − 1)/2. The topological density (TD_10_) of the net was calculated as the sum of all the vertices in the first 10 coordination shells of the initial vertex of the net.

In order to assess the relative complexities of the crystal structures, the implicit hierarchical depth (IHD) of a crystal structure was estimated. In contrast to the explicit HD [60], IHD equals the smallest number of equivalence classes of the edges linking the ions into a simply connected net; in other words, IHD represents the number of bearing contacts of the structure. IHD should be classified as an assembly index, which shows how many steps one needs to take before a specific composite object can arise from basic building blocks [61]. Being a generalization of the critical coordination number introduced for 0D b.u. [30], IHD is relevant to any structure with any dimensionality and periodicity of its b.u. In the ordinary crystallographic context, IHD is evaluated with respect to 0D b.u. of the crystal structure and thus depends on the space group and the list of Wyckoff positions (WP) occupied by the b.u., viz.:

IHD ≡ inf(*e″*) = |*U*_SG_| + *Z″* − 1 − *f*(WP_1_, WP_2_, ..., WP*_Z″_*),

where *e″* is the number of equivalence classes of edges in the simply connected net; |*U*_SG_| is the number of elements in any minimal generating subset of the space group; *Z″* is the total number of orbits occupied by the b.u.; and the parameter *f*(WP_1_, WP_2_, ..., WP*_Z″_*) is influenced by the site-symmetry groups of occupied positions. In the simplest case, when the b.u. occupy only general positions, *f* = 0.

## Figures and Tables

**Table 1:** Crystallographic data for compounds belonging to the (enH_2_)[*M*(H_2_O)_4_(*TX*_4_)_2_] structural family.

Compound	Space Group	Unit Cell Parameters			Ref.
		*a*, Å α, °	*b*, Å β, °	*c*, Å γ, °	
(enH_2_)[Mg(H_2_O)_4_(SO_4_)_2_]	*P*-1	6.7847(4) 74.909(2)	7.0721(4) 72.378(2)	7.2217(4) 79.564(3)	[18]
(enH_2_)[Mn(H_2_O)_4_(SO_4_)_2_]	*P*-1	6.850(2) 74.39(2)	7.188(2) 72.02(2)	7.304(2) 78.79(2)	[19]
(enH_2_)[Fe(H_2_O)_4_(SO_4_)_2_]	*P*-1	6.8350(3) 75.012(4)	7.1253(3) 72.355 (4)	7.2235(4) 79.185(4)	[20]
(enH_2_)[Co(H_2_O)_4_(SO_4_)_2_]	*P*-1	6.8033(2) 74.909(2)	7.0705(2) 72.291(2)	7.2192(3) 79.167(2)	[21,35]
(enH_2_)[Cd(H_2_O)_4_(SO_4_)_2_]	*P*-1	6.9114(2) 74.013(2)	7.3056(2) 71.731(1)	7.3629(1) 78.043(1)	[22]
(enH_2_)[Cu(H_2_O)_4_(SO_4_)_2_]	*P*2_1_/*c*	5.152(2)	11.636(4) 94.80(1)	10.219(4)	[17]
(enH_2_)[Cu(H_2_O)_4_(BeF_4_)_2_]	*P*2_1_/*c*	5.1044(2)	11.6171(4) 94.431(3)	10.1178(3)	This work
(enH_2_)[Cu(H_2_O)_4_(SeO_4_)_2_]	*P*2_1_/*c*	5.2502(1)	11.7500(2) 94.5464(17)	10.4434(2)	This work

**Table 2:** Crystallographic data and refinement parameters for compounds **1** and **2**.

Compound	1	2
Chemical formula	(C_2_N_2_H_10_)Cu(H_2_O)_4_(BeF_4_)_2_	(C_2_N_2_H_10_)Cu(H_2_O)_4_(SeO_4_)_2_
Symmetry	Monoclinic	Monoclinic
Space group	*P*2_1_/c	*P*2_1_/*c*
Unit cell parameters		
*a,* Å	5.1044(2)	5.2502(1)
*b*, Å	11.6171(4)	11.7500(2)
*c,* Å	10.1178(3)	10.4434(2)
*β,* °	94.431(3)	94.5464(17)
*V*, Å^3^	598.18(4)	642.22(2)
*Z*	2	2
*D_x_* (g cm^−3^)	2.0417	2.501
Diffractometer	Rigaku XtaLAB Synergy (HyPix detector)	
Radiation	Mo*K*α, *l* = 0.71073 Å	
Data range θ, °	3.51–30.85	3.47–30.75
Limits: *h*, *k*, *l*	−6 < *h* < 7, −15 < *k* < 16, −13 < *l* < 12	−7 < *h* < 6, −16 < *k* < 16, −14 < *l* < 12
No. of measured reflections	7823	8380
No. of total reflections, *R*_int_	1524, 0.0377	1573, 0.0256
No. of observed reflections, *I >* 3*σ*(*I*)	1235	1438
*R*_1_ [*I* > 3σ(*I*)], *wR*_2_ [*I* > 3σ(*I*)]	0.0214, 0.0225	0.0164, 0.0183
*R*_1_ [all], *wR*_2_ [all]	0.0298, 0.0237	0.0193, 0.0190
GoF on *F*^2^	1.07	1.19
CCDC	2,390,641	2,390,642

**Table 3:** The assignments of the Raman bands in the spectra of compounds of the (enH_2_)[Cu(H_2_O)_4_(*TX*_4_)_2_] structural family.

*TX*_4_ = BeF_4_	*TX*_4_ = SO_4_	*TX*_4_ = SeO_4_	Assignments
**288** 333	297	217 288 332	**ν_2_ (BeF_4_^2−^)**; Deformation modes of [Cu(H_2_O)_4_]^2+^; NCCN def
**359**	361	**360**	**ν_4_ (BeF_4_^2−^), ν_2_ (SeO_4_^2−^)** CN tors
430	**449**	417 440	**ν_2_ (SO_4_^2−^), ν_4_ (SeO_4_^2−^)** Valence modes of Cu(H_2_O)_4_
479	480	472	
	**612, 630**		**ν_3_ (SO_4_^2−^)**
**827**	820	**824**	**ν_3_ (BeF_4_)**, **ν_1_ (SeO_4_)**
875	879	872 [ν_3_ (SeO_4_)]	R (H_2_O)
**932**	934	930	**ν_1_ + ν_4_ (BeF_4_^2−^)**
1062	**977**	958	ν (C-N); **ν_1_ (SO_4_^2−^)**
	1014 **1066** 1123	1061	**ν_3_ (SO_4_^2−^)**
1233	1235	1238	ν_2_ (H_2_O)
1245	1247		t(NH_3_)
1341	1341	1339	t(NH_3_),
1476	1481	1475	
1615	1604	1591 1614	δ(CH_2_) ν_2_ (H_2_O)
2844	2847	2845	ν(CH_2_)
2934	2932	2930	ν(CH_2_),
2958 2989	2967 2996	2957 2988	
3143	3156	3159	

The assignments to the individual anions are highlighted in bold.

## Data Availability

No new data was created.

## Supplementary Materials

The following supporting information can be downloaded at https://www.mdpi.com/article/10.3390/molecules29225372/s1: Table S1: Selected bond distances (Å) in the crystal structures of 1, 2, and (C_2_N_2_H_10_)Cu(H_2_O)_4_(SO_4_)_2_; Table S2: The geometrical features of hydrogen bonds in the crystal structures of 1, 2 and (C_2_N_2_H_10_)Cu(H_2_O)_4_(SO_4_)_2_.
