# Supplementary material for: The (C2N2H10)[Cu(H2O)4](TX4)2 Structural Family: When Fluoroberyllate, Sulfate, and Selenate Are Full Analogs
Source: Molecules. 2024 Nov 14;29(22):5372. doi: 10.3390/molecules29225372 (PMC11596614; doi:10.3390/molecules29225372)
Supplement: Supplementary file 1 [file molecules-29-05372-s001.zip › molecules-3284254-supplementary/molecules-3284254 - supplementary.pdf]

Supplementary Materials

Table S1. Selected bond distances (Å) in the crystal structures of 1, 2 and $(C_2N_2H_{10})Cu(H_2O)_4(SO_4)_2$.

Structure	$(C_2N_2H_{10})Cu(H_2O)_4(BeF_4)_2$	$(C_2N_2H_{10})Cu(H_2O)_4(SO_4)_2$*	$(C_2N_2H_{10})Cu(H_2O)_4(SeO_4)_2$
	BeF_4	SO_4	SeO_4
bond distances, Å			
average T-X	1.551(2)	1.477(2)	1.641(1)
range of T-X	1.536(2) - 1.567(2)	1.464(2) - 1.487(2)	1.633(1) - 1.649(1)
bond angles, deg			
average X-T-X	109.48(10)	109.47(6)	109.47(6)
range of X-T-X	106.37(10) - 112.49(10)	110.35(6) - 110.02(6)	108.25(5) - 111.12(6)
	$C_2N_2H_{10}$	$C_2N_2H_{10}$	$C_2N_2H_{10}$
bond distances, Å			
average C-N	1.483(2)	1.482(2)	1.484(2)
average C-C	1.521(2)	1.511(2)	1.516(2)
bond angles, deg			
average C-C-N	108.76(10)	108.98(10)	108.50 (11)
	$Cu(H_2O)_4F_2$	$Cu(H_2O)_4O_2$	$Cu(H_2O)_4O_2$
bond distances, Å			
average Cu-OH_2	1.940(2)	1.940(2)	1.949(2)
range of Cu-OH_2	1.931(2) - 1.948(2)	1.938(2) - 1.941(2)	1.945(2) - 1.952(1)
average Cu-X	2.592(2)	2.671(2)	2.620(1)
bond angles, deg			
average H_2O-Cu-OH_2	90	90	90
range of H_2O-Cu-OH_2	86.56(5) - 93.44(5)	83.06(4) - 96.94(4)	85.81(5) - 94.19(5)

average T-Cu-T	179.99(3)	179.99(3)	180.00(3)
average H_2O-Cu-T	90	90	90
range of H_2O-Cu-T	83.34(4) - 96.66(4)	83.34(4) - 96.66(4)	82.42(4) - 97.58(4)

*data were taken from [13].

Table S2. The geometrical features of hydrogen bonds in the crystal structures of 1, 2 and $(C_2N_2H_{10})Cu(H_2O)_4(SO_4)_2$.

$(C_2N_2H_{10})Cu(H_2O)_4(BeF_4)_2$			
	angle, deg	distance, Å	
	N-H --- F	N---F	H---F
N(1)-H(8) --- F(1)	166.54(194)	2.759(5)	1.91(2)
N(1)-H(9) --- F(2)	119.73(180)	2.575(1)	2.376(21)
N(1)-H(7) --- F(3)	160.00(189)	2.791(4)	1.944(21)
N(1)-H(9) --- F(4)	152.23(206)	2.845(2)	2.114(21)
	O-H --- F	O---F	H---F
O(1)-H(2) --- F(1)	173.23(213)	2.609(3)	1.847(21)
O(1)-H(1) --- F(2)	172.09(236)	2.677(3)	1.828(22)
O(2)-H(4) --- F(3)	163.97(208)	2.679(2)	1.93(2)
O(2)-H(3) --- F(4)	167.60(238)	2.616(3)	1.813(25)
$(C_2N_2H_{10})Cu(H_2O)_4(SO_4)_2$ [13]			
	angle, deg	distance, Å	
	N-H --- O	N---O	H---O
N(1)-H(6) --- O(1)	117.16(172)	2.834(5)	1.929(20)
N(1)-H(3) --- O(2)	119.73(180)	2.905(3)	2.370(22)
N(1)-H(4) --- O(3)	164.04(194)	2.882(4)	2.056(22)
N(1)-H(6) --- O(4)	146.38(209)	2.899(2)	2.091(24)
	O-H --- O	O---O	H---O
O(5)-H(9) --- O(1)	168.27(238)	2.688(2)	1.922(23)
O(5)-H(10) --- O(2)	167.22(226)	2.677(3)	1.831(24)
O(6)-H(7) --- O(3)	167.16(310)	2.747(3)	2.031(29)
O(6)-H(8) --- O(4)	169.90(225)	2.652(3)	1.733(25)
$(C_2N_2H_{10})Cu(H_2O)_4(SeO_4)_2$			
	angle, deg	distance, Å	
	N-H --- O	N---O	H---O
N(1)-H(5) --- O(1)	167.34(190)	2.810(3)	1.88(2)

N(1)-H(8) --- O(2)	162.66(185)	2.895(2)	2.346(22)
N(1)-H(8) --- O(4)	147.30(209)	2.957(2)	2.129(24)
N(1)-H(6) --- O(6)	158.88(225)	2.884(3)	2.151(25)
	O-H --- O	O---O	H---O
O(3)-H(3) --- O(4)	167.27(258)	2.673(2)	1.924(26)
O(3)-H(9) --- O(6)	171.84(235)	2.758(2)	2.091(21)
O(5)-H(2) --- O(1)	168.39(238)	2.686(2)	1.892(24)
O(5)-H(1) --- O(2)	170.86(241)	2.685(2)	1.914(23)
